# Therapeutic efficacy and effects of artemisinin-based combination treatments on uncomplicated *Plasmodium falciparum* malaria -associated anaemia in Nigerian children during seven years of adoption as first-line treatments

**DOI:** 10.1186/s40249-016-0217-7

**Published:** 2017-02-07

**Authors:** Akintunde Sowunmi, Kazeem Akano, Godwin Ntadom, Adejumoke I. Ayede, Folasade O. Ibironke, Temitope Aderoyeje, Elsie O. Adewoye, Bayo Fatunmbi, Stephen Oguche, Henrietta U. Okafor, Ismaila Watila, Martin Meremikwu, Philip Agomo, William Ogala, Chimere Agomo, Onikepe A. Folarin, Grace O. Gbotosho, Christian T. Happi

**Affiliations:** 1 Department of Pharmacology and Therapeutics, University of Ibadan, Ibadan, Nigeria; 2 Institute for Medical Research and Training, College of Medicine, University of Ibadan, Ibadan, Nigeria; 3 National Malaria Elimination Programme, Federal Ministry of Health, Abuja, Nigeria; 4 Department of Paediatrics, University of Ibadan, Ibadan, Nigeria; 5 Department of Clinical Pharmacology, University College Hospital, Ibadan, Nigeria; 6 Department of Physiology, University of Ibadan, Ibadan, Nigeria; 7 World Health Organization, Regional Office for the Western Pacific, Khan Daun Penh, Phnom Penh Cambodia; 8 Department of Paediatrics, University of Jos, Jos, Nigeria; 9 Department of Paediatrics, Institute of Child Health, University of Nigeria Teaching Hospital, Enugu, Nigeria; 10 Department of Paediatrics, Specialist Hospital, Maiduguri, Borno Sate Nigeria; 11 Department of Paediatrics, University of Calabar, Calabar, Cross Rivers State Nigeria; 12 Nigeria Institute of Medical Research, Yaba, Lagos, Nigeria; 13 Department of Paediatrics, Ahmadu Bello University, Zaria, Nigeria; 14 Department of Biological Sciences, Redeemer’s University, Ede, Osun State Nigeria

**Keywords:** Malaria-associated anaemia, “Haematocrit conservation”, Artemisinin-based combination treatments, Children, Nigeria

## Abstract

**Background:**

Artemisinin-based combination treatments (ACTs) are the first-line treatments of uncomplicated *Plasmodium falciparum* malaria in many endemic areas but there are few evaluation of their efficacy in anaemic malarious children.

**Methods:**

Therapeutic efficacy of 3-day regimens of artesunate-amodiaquine and artemether-lumefantrine was evaluated in 437 anaemic and 909 non-anaemic malarious children following treatment during a seven-year period (2008–2014). Patterns of temporal changes in haematocrit were classified based on haematocrit values <30% and ≥30%. Kinetics of the disposition of the deficit in haematocrit from 30% following treatment were evaluated using a non-compartment model.

**Results:**

PCR-corrected parasitological efficacy 28 days after start of treatment was significantly higher in artesunate-amodiaquine- compared to artemether-lumefantrine-treated children [97% (95%*CI*: 92.8–100) versus 96.4% (95%*CI*: 91.3–99.4), *P* = 0.02], but it was similar in non-anaemic and anaemic children. Fall in haematocrit/1 000 asexual parasites cleared from peripheral blood was significantly greater at lower compared to higher parasitaemias (*P* < 0.0001), and in non-anaemic compared to anaemic children (*P* = 0.007). In anaemic children at presentation, mean anaemia recovery time (AnRT) was 15.4 days (95%*CI*: 13.3–17.4) and it did not change over the years. Declines in haematocrit deficits from 30% were monoexponential with mean estimated half-time of 1.4 days (95%*CI*: 1.2–1.6). Anaemia half-time (t_½anaemia_) correlated positively with AnRT in the same patients (*r* = 0.69, *P* < 0.0001). Bland-Altman analysis of 10 multiples of t_½anaemia_ and AnRT showed narrow limit of agreement with insignificant bias (*P* = 0.07) suggesting both can be used interchangeably in the same patients.

**Conclusions:**

Artesunate-amodiaquine and artemether-lumefantrine remain efficacious treatments of uncomplicated *P. falciparum* infections in non-anaemic and anaemic Nigerian children in the last 7 years of adoption as first-line treatments. These ACTs may also conserve haematocrit at high parasitaemias and in anaemic children.

**Trials registration:**

Pan African Clinical Trial Registry PACTR201508001188143, 3 July 2015; PACTR201510001189370, 3 July 2015; PACTR201508001191898, 7 July 2015 and PACTR201508001193368, 8 July 2015.

**Electronic supplementary material:**

The online version of this article (doi:10.1186/s40249-016-0217-7) contains supplementary material, which is available to authorized users.

## Multilingual abstracts

Please see Additional file 1 for translations of the abstract into the six official working languages of the United Nations.

## Background

Recommended as the first-line treatments of uncomplicated *Plasmodium falciparum* malaria globally [1], artemisinin-based combination treatments (ACTs) have remained largely efficacious globally except in the Greater Mekong subregion where artemisinin resistance in *P. falciparum* has recently emerged [2–7]. Not only do these drug combinations clear asexual and immature sexual parasitaemia rapidly and prevent progression of committed and non-committed asexual parasites to sexual forms, they may also prevent destruction of once-parasitized (once-infected) red blood cells through a splenic process called “pitting”. Pitting removes the dead parasites from parasitized red blood cells and returns the once-infected red blood cells into circulation [8–10]. This process prevents precipitous falls in haematocrit in the first few days following ACTs particularly when parasitaemias are high. In severe malaria, pitting is a life-saving process [10].

It has been suggested that in resource-poor endemic countries, the degree of precipitous falls in haematocrit following ACTs can be measured by estimating the fall in haematocrit per 1 000 red blood cells cleared from peripheral blood in the first two days following treatment [11]. The relatively little or no fall in baseline (pre-treatment) haematocrit in the first few days following treatment, particularly when parasitaemias are high, has been termed “haematocrit conservation” [11].

In many endemic and non-endemic areas of the world, anaemia is an inevitable consequence of untreated *P. falciparum* infections. Anaemia may occur in 10 – 90% of children or non-immune individuals presenting with acute infections [12–18]. Malaria-associated anaemia contributes significantly to morbidity or mortality in *P. falciparum* malaria [19–25]. Despite the frequent occurrence of malaria-associated anaemia in children living in endemic areas, the efficacy of artemisinin-based combination treatments and the adverse events following their use have been little evaluated in anaemic children with uncomplicated *P. falciparum* infections.

It has recently been reported that intravenous artesunate treatment may cause delayed haemolysis in immunologically naïve patients with severe malaria [10, 26–31]. However, it is unclear if artemisinin-based combination treatments conserve haematocrit in anaemic children following treatment of uncomplicated *P. falciparum* infections. It is also unclear if the conserved haematocrit is subsequently lost resulting in a late-appearing anaemia in children with uncomplicated infections.

In Nigeria, artemether-lumefantrine and artesunate-amodiaquine, in that order, were adopted as first-line treatments of uncomplicated *P. falciparum* malaria in 2005 [32]. Both ACTs have been evaluated, using standardised protocols, more or less continuously at one of seven sentinel sites set up by Nigeria’s Federal Ministry of Health in six geographical areas of Nigeria. These sentinel sites were set up to monitor the efficacy of antimalarial drugs. There is no reported study, in Nigerian children, of the efficacy of artemether-lumefantrine and artesunate-amodiaquine in the last seven years of their adoption as first-line treatments.

The aims of the present study during a 7-year period of adoption are: (i) to evaluate the efficacy of artesunate-amodiaquine and artemether-lumefantrine in uncomplicated *P. falciparum* malaria, (ii) to determine if efficacy of artesunate-amodiaquine and artemether-lumefantrine differs between malarious anaemic and malarious non-anaemic children, and if ACTs conserve haematocrit in anaemic children, (iii) to evaluate recovery from malaria-associated anaemia, and (iv) to elucidate the temporal changes in haematocrit following treatment with artesunate-amodiaquine and artemether-lumefantrine in anaemic malarious children.

## Methods

### Study locations

**Fig. 1:**
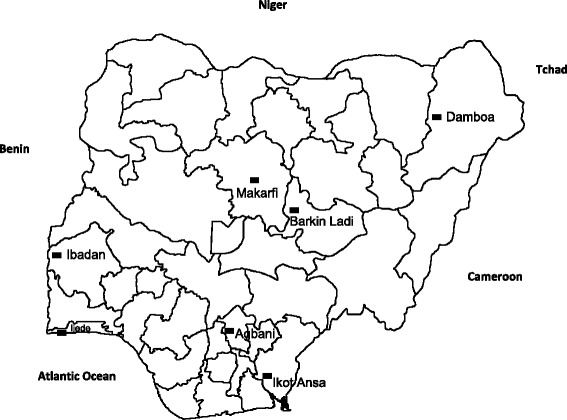
Map of Nigeria showing study locations in seven states

**Fig. 2:**
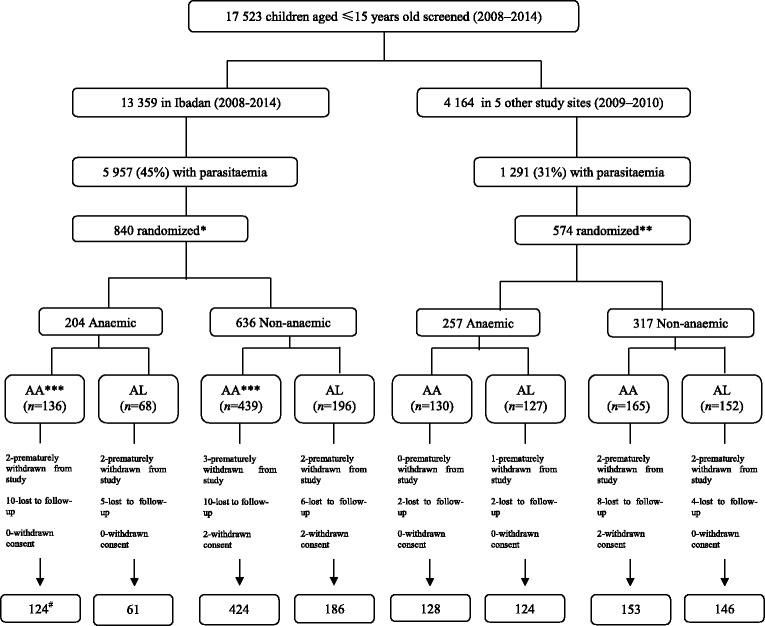
Study profile. AA, artesunate-amodiaquine; AL, artemether-lumefantrine, * follow-up was for 42 days, ** follow-up was for 28 days, *** randomization at a ratio of 2:1 for artesunate-amodiaquine and artemether-lumefantrine, # number of children that completed follow-up period, ^ children are <5 years old.

The studies were part of a programme to monitor antimalaria therapeutic efficacy at seven sentinel sites located in six geographical areas of Nigeria (Fig. 1). These sites were established by Nigeria’s Federal Ministry of Health. These studies were conducted between October 2009 and November 2010 at the following locations: Agbani, Ikot Ansa, Barkin Ladi and Damboa, in Enugu, Cross River, Plateau and Borno States, respectively (the eastern flank of the study sites), and in Ijede, and Makarfi in Lagos, and Kaduna States, respectively (the western flank). The studies were also conducted continuously in Sabo quarters of Ibadan, Oyo State (the reference centre), located on the western flank from 2008 to 2014 (Fig. 1). In virtually all study locations, malaria is endemic and transmission occurs all year round; however, it is more intense during the rainy season from April to October. *P. falciparum* is the predominant species, accounting for over 98% of all infections [33, 34]. Children are more affected than adults, and apparently, asymptomatic infections occur in older school children and adults [33]. The overall study profile is shown in Fig. 2.

### Study procedures

Standardised procedures and protocol were used at all sentinel sites [35–40]. Briefly, patients were eligible to participate in the study if they were: aged 6 months–15 years, had symptoms compatible with acute uncomplicated malaria with *P. falciparum* mono-infections ≥1 000 μL^−1^ of blood, no history of antimalarial drug ingestion in the two weeks prior to enrolment, absence of severe malaria and written informed consent given by parents or guardians.

Enrolled patients were randomized to receive artemether-lumefantrine or artesunate-amodiaquine (co-formulated) for 3 days (days 0–2) as previously described [35, 40]. The day of presentation (day of starting treatment) was regarded as day 0. Thick and thin blood films were obtained from each child as soon as they came to the clinic and the slides were carefully labelled with the patients’ codes and air-dried before being stained. Follow-up with clinical and parasitological evaluation was done daily on days 1–3 and on days 7, 14, 21, 28 at all study sites except in Ibadan where additional follow-up was done on days 35 and 42. Parasitaemia, asexual or sexual, in thick films was estimated by counting asexual and sexual parasites relative to 500 leukocytes, or 500 asexual or sexual forms whichever occurred first. From this figure, the parasite density was calculated assuming a leukocyte count of 6 000 μL^−1^ of blood [41–43]. Sexual parasitaemia was estimated only in Ibadan, but their presence or absence was recorded at other sites. A slide was considered asexual parasite negative if no asexual parasite was detected after examination of 200 microscope fields.

The cure rates on days 28 and 42 were adjusted on the basis of the polymerase chain reaction (PCR) genotyping results of paired samples of patients with recurrent parasitaemia after day 7 of starting treatment using the World Health Organisation 2003 and 2009 protocols [44, 45] as previously described [35–38]. The clinical classification system consisted of the following categories of response: adequate clinical and parasitological response (ACPR), late parasitological failure (LPF), late clinical failure (LCF), early treatment failure (ETF). The primary outcomes were the 28-day uncorrected and PCR-corrected efficacy. Asexual parasite reduction ratio (PRR) [46] was defined as the ratio of day 0/day 2 parasitaemia (and for convenience, referred to as PRR_D2_). If there was complete clearance of parasitaemia on day 2, parasitaemia was assumed to be 1 uL^−1^, a level below microscopic detection. Asexual parasite reduction ratio on day 1 (PRR_D1_) was defined as the ratio of day 0/day 1 parasitaemia. If there was complete clearance of parasitaemia on day 1, parasitaemia was assumed to be 1 uL^−1^, a level below microscopic detection.

#### Haematological evaluation

**Fig. 3:**
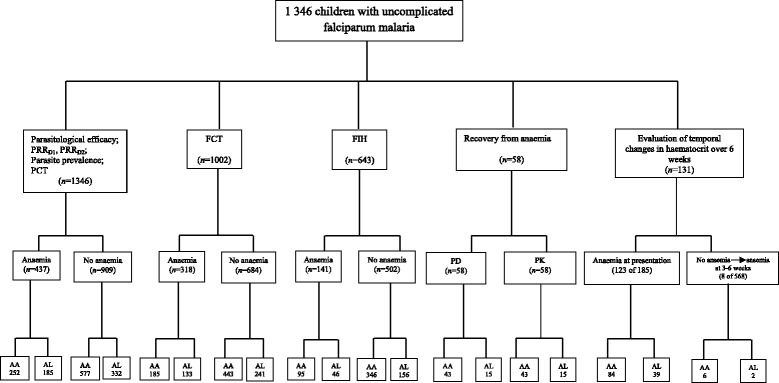
Profile of investigations carried out. PRR_D1_, parasite reduction ratio 1 day after treatment began; PRR_D2_, parasite reduction ratio 2 days after treatment began; PCT, parasite clearance time; FCT, fever clearance time; FIH, fall in haematocrit per 1 000 asexual parasites cleared from peripheral blood; PD, pharmacodynamic measure of recovery (anaemia recovery time); PK, pharmacokinetic measure of recovery (anaemia half-time); AA, artesunate-amodiaquine; AL, artemether-lumefantrine

Capillary blood collected before and during follow-up was used to measure haematocrit using a microhaematocrit tube and microcentrifuge (Hawksley, Lancing, UK). Anaemia was defined as a haematocrit <30% and was classified as mild, moderate or severe if haematocrit was 21-29%, 15-20% or <15%, respectively. Anaemia recovery time (in anaemic patients) was defined as time elapsing from drug administration to attainment of a haematocrit value ≥30% and was evaluated in children with haematocrit ≤25% at presentation. In patients who had early or late monophasic declines in haematocrit which resulted in anaemia, anaemia recovery time was defined as time from appearance of, to recovery from, anaemia. Fall in haematocrit per 1 000 asexual parasites cleared from peripheral blood following treatment (FIH/1 000 asexual parasites cpb) was defined as numeric estimation of relative difference in haematocrit at baseline (pre-treatment) and the first 1 or 2 days after treatment began as numerator, and the corresponding relative difference in parasitaemia as the denominator, and expressing it per 1 000 asexual parasites cleared from peripheral blood $\left[\frac{FIH}{1000} asexual\kern0.5em  parasite\kern0.5em cpb\kern0.5em =\kern0.5em \frac{Haematocrit\kern0.5em  on\kern0.5em  day\kern0.5em 0- Haematocrit\kern0.5em  on\kern0.5em  day\kern0.5em 1\kern0.5em  or\kern0.5em 2}{Parasitaemia\kern0.5em  on\kern0.5em  day\kern0.5em 0- Parasitaemia\kern0.5em  on\kern0.5em  day\kern0.5em 1\kern0.5em  or\kern0.5em 2}\times 1000\right]$ [11]. Fig. 3 is the profile of investigations carried out during the study.

#### Evaluation of temporal changes in haematocrit in anaemic children following treatment

Haematocrit <30% and ≥30% were the reference points in all classified patterns and was modified from a recently described patterns [47]. Temporal changes in haematocrit were classified into the following patterns.

1. Haematocrit <30% before treatment followed by an increase to ≥30% after treatment (malaria-associated anaemia at presentation and recovery from anaemia).
2. Haematocrit <30% followed by a rise to ≥30% by day 7, a fall to <30% between days 7 and 14 and then recovery (anaemia–early recovery–anaemia–late recovery).
3. Haematocrit <30% followed by a rise to ≥30% on day 7 or 14, followed by two consecutive normal haematocrits and decline to <30% after day 14 (anaemia–early recovery–late-appearing anaemia pattern).
4. Haematocrit <30% before treatment began and during the entire follow-up period (persistent, unresolved anaemia).
5. Multiple falls in haematocrit below 30%, a rise to ≥30% during follow-up period and then a fall below 30% (undulating pattern of anaemia).
6. Unclassifiable.

In non-anaemic patients (*n* = 568), evaluation of temporal changes was limited to those who had falls in haematocrit to anaemic level 3–6 weeks after commencement of treatment as previously described [47].

#### Kinetics of the disposition of deficit in haematocrit from 30%

The kinetics of the disposition of deficit in haematocrit from 30%, that is, of anaemia, was as previously described [35, 37, 47]. Briefly, in all anaemic patients with a haematocrit value ≤25% at enrolment, or when anaemia occurs following treatment, haematocrit values below 30% (the lower threshold of normal) and at follow-up were subtracted from 30% at each time of measurement until haematocrit rose to ≥30%, and the resulting values plotted against time. The final haematocrit when anaemia resolved was therefore zero in all patients. However, the final haematocrit at the time of resolution or recovery was assumed to be 0.01%. The areas under the curve (AUC) of deficit in haematocrit (from 30%) *versus* time were obtained, by trapezoidal rule using the computer program *Turbo Ken* (designed by Clinical Pharmacology Group, University of Southampton, United Kingdom) as previously described [35, 37]. AUC was also obtained manually by calculating the average haematocrit values between two consecutive time measurements and multiplying it by the time interval between the measurements, and summing up all the values, in a manner similar to that for the numerical estimation of area under a drug concentration-time curve [48]. The unit of quantification would be %.d, if haematocrit values were used or g/L.d if haemoglobin values were used. Haematocrit values may be converted to haemoglobin values by dividing by 3 [49]. Semilog plots of deficit in haematocrit *versus* time were plotted. The apparent terminal elimination rate constant (λ) was obtained by least-square regression analysis of the post-peak log-linear part of the plot of deficit in haematocrit (from 30%) versus time, and the apparent terminal half-time of anaemia (t_1/2(anaemia)_) was calculated from ln 2/(λ) (that is, λt = 0.693).

#### Statistical analysis

Data were analysed using version 6 of Epi-Info software [50] and the statistical program SPSS for Windows version 20.0 [51]. Variables considered in the analysis were related to the densities of *P. falciparum* asexual and sexual forms. Proportions were compared by calculating *χ* ^2^ using Yates’ correction, Fisher’s exact or Mantel Haenszel tests. Normally distributed, continuous data were compared by Student’s *t* test and analysis of variance (ANOVA). Data not conforming to a normal distribution were compared by the Mann–Whitney U tests and the Kruskal Wallis tests (or by Wilcoxon ranked sum test). The cumulative risk of parasite reappearance was calculated by survival analysis using Kaplan-Meier method. Correlation between anaemia recovery time and anaemia half-time in the same patients was assessed by Pearson’s correlation coefficient. Agreement between multiples of anaemia half-time (proposed pharmacokinetic method of assessing response to treatment) and anaemia recovery time (standard pharmacodynamic method of assessing response to treatment) in the same patients was assessed by Bland-Altman analysis [52]. Impacts of treatments over time were evaluated using test for trend for the following parameters: parasitological efficacy, late parasitological failure and gametocyte carriage, and by comparison of mean or median at specific time intervals for the following parameters: parasite reduction ratios 1 or 2 days after treatment began, parasite clearance time, fever clearance time, median FIH/1 000 asexual parasite cpb and anaemia recovery time. *P* values of <0.05 were taken to indicate significant differences. Data were double entered serially using patients’ codes and were only analysed at the end of the study.

### Ethical clearance

The study protocol was approved by the Ministry of Health, Ibadan, and the National Health Research Ethics Committee, Abuja, Nigeria. The reference numbers are: Ministry of Health Ibadan - AD 13/262/56 (7 March 2006), AD 13/479/978 (December 2015); National Health Research Ethics Committee - NHREC/01/01/2007-28/10/2009d (30 October 2009), NHREC/01/01/2007-28/10/2013 (29 October 2013), NHREC/01/01/2007-22/10/2014 (30 October 2014). Written informed consents were obtained from parents/guardians of the children.

## Results

### Patient characteristics at enrolment

During the study period, 1 346 children of which 437 children (32%) who were anaemic at presentation were included in the present study (Fig. 3). A total of 829 and 517 children were treated with artesunate-amodiaquine and artemether-lumefantrine, respectively. Of the 437 anaemic children, anaemia was mild, moderate or severe in 395 (90.4%), 40 (9.1%) or 2 (0.5%) children, respectively. Overall, the mean age of these children was 5.3 years (95%*CI*: 5.1-5.5, range 0.5-15). The clinical and parasitological characteristics of these children are summarised in Table 1. Overall (see All treatments section in Table 1), children with anaemia were significantly younger, had significantly longer duration of illness and a significantly lower enrolment parasitaemia.

**Table 1:** Baseline characteristics of children treated with artesunate-amodiaquine or artemether-lumefantrine between 2008 and 2014

	Artesunate-amodiaquine (829)		*P* value	Artemether-lumefantrine (517)		*P* value	All treatments (1346)		*P* value
	No anaemia (577)	Anaemia (252)		No anaemia (332)	Anaemia (185)		No anaemia (909)	Anaemia (437)	
Gender (M/F)	300/277	141/111	0.29	183/149	102/83	1.00	483/426	243/194	0.39
Age (year)^a^									
Mean	6.4	4.3	<0.0001	5	3.9	<0.0001	5.9	4.1	<0.0001
95%*CI*	6.2-6.7	4.0-4.7		4.6-5.3	3.5-4.2		5.7-6.1	3.9-4.4	
No. < 5 years	246	183	<0.0001	219	156	<0.0001	465	339	<0.0001
Duration of illness (day)									
Mean	2.7	3.3	<0.000	2.9	3.2	0.03	2.8	3.3	<0.0001
95%*CI*	2.6-2.9	3.01-3.5	1	2.7-3	2.9-3.6		2.7-2.9	3.1-3.5	
Temperature (°C)									
Mean	38.2	38.1	0.16	38.1	38.	0.35	38.2	38.1	0.08
95%*CI*	38.1-38.2	37.9-38.2		38-38.3	37.9-38.2		38.1-38.3	38.0-38.2	
No. > 37.4 °C	443	185	0.30	241	133	0.87	684	318	0.33
No. > 40 °C	25	18	0.08	13	13	0.12	38	31	0.02
Haematocrit (%)									
Mean	33.9	25.6	<0.0001	33.5	25.5	<0.0001	33.8	25.5	0.009
95%*CI*	33.7-34.2	25.24 – 26.0		33.2-33.8	25.1-26		33.6-34	25.3-25.8	
No. < 30%		252			185			437	
Parasitaemia (μL^−1^)									
Geometric mean	38 917	23 908	<0.0001	27 791	20 512	0.03	34 413	22 407	<0.0001
Range	1 000–1 125 000	1 000–1 096 636		1 000–1 000 000	1 000–2 124 000		1 000–1 125 000	1 000–2 124 000	
No. ≥ 100 000 μL^−1^	141	52	0.27	67	26	0.08	208	78	0.04
No. ≥ 250 000 μL^−1^	35	8	0.12	13	5	0.47	48	13	0.06

^a^Mean age for all children enrolled in the study is 5.3 years (95%*CI* 5.1-5.5, range 0.5-15, *n* = 1 346)

### Therapeutic responses

#### Primary outcomes

##### Parasitological efficacy

Overall, during the 7-year period, parasitological efficacy (ACPR) on day 28 with both treatments was 96.5% (95%*CI*: 91.8-100) and it increased significantly with time over the study period [94.6% (95%*CI*: 84.1-100) *versus* 98.8% (95%*CI*: 84.0-100) *P* = 0.007 test for trend, in 2008–2010 and 2011–2014, respectively]. Overall, parasitological efficacy on day 28 was significantly higher in children treated with artesunate-amodiaquine compared with children treated with artemether-lumefantrine [97% (95%*CI*: 92.8-100) *versus* 96.4% (95%*CI*: 91.3-99.4); *P* = 0.02)]. The significant increase in parasitological efficacy over the years involved both artesunate-amodiaquine and artemether-lumefantrine and both anaemic and non-anaemic children (data not shown).

Overall, parasitological efficacy was similar in anaemic and non-anaemic children (97.5% (95%*CI*: 92.4-100) *versus* 96.1% (95%*CI*: 91.2-100), *P* = 0.3, respectively). In children treated with artesunate-amodiaquine, PCR-uncorrected and corrected parasitological efficacy were similar in anaemic and non-anaemic children at all study sites (Table 2). Similarly, in children treated with artemether-lumefantrine, PCR-uncorrected and corrected parasitological efficacy were similar in anaemic and non-anaemic children at all sites (Table 2).

**Table 2:** Efficacy of artesunate-amodiaquine or artemether-lumefantrine in anaemic and non-anaemic malarious children according to study site and year of enrolment

Site (Year)	Haematocrit status	PCR uncorrected			PCR corrected			
		ACPR_u	Failure_u	Total	ACPR_c	Recrudescence	Total	*P* value
Artesunate-amodiaquine								
Ibadan (2008–2010)	Anaemia	84	5	89	85	3	88	
	No anaemia	286	14	300	286	11	297	1.0
Damboa (2009–2010)	Anaemia	40	0	40	40	0	40	
	No anaemia	21	0	21	21	0	21	-
Agbani (2009–2010)	Anaemia	26	5	31	25	5	30	
	No anaemia	21	2	23	20	2	22	0.44
Makarfi (2009–2010)	Anaemia	22	0	22	22	0	22	
	No anaemia	39	0	39	39	0	39	-
Ijede (2009–2010)	Anaemia	19	1	20	19	1	20	
	No anaemia	28	0	28	28	0	28	-
Barkin Ladi (2009–2010)	Anaemia	11	4	15	11	0	11	
	No anaemia	36	6	42	36	4	40	-
Ibadan (2011–2014)	Anaemia	34	1	35	34	0	34	
	No anaemia	121	3	124	119	4	123	-
Total (2008–2014)	Anaemia	236	16	252	236	9	245	
	No anaemia	552	25	577	549	21	570	0.85
Artemether-lumefantrine								
Ibadan (2008–2010)	Anaemia	43	2	45	43	2	45	
	No anaemia	129	11	140	132	8	140	1.0
Damboa (2009–2010)	Anaemia	33	3	36	33	2	35	
	No anaemia	18	2	20	18	1	19	1.0
Agbani (2009–2010)	Anaemia	33	2	35	33	0	33	
	No anaemia	19	4	23	19	4	23	-
Makarfi (2009–2010)	Anaemia	20	1	21	19	1	20	
	No anaemia	37	0	37	37	0	37	-
Ijede (2009–2010)	Anaemia	19	1	20	19	0	19	
	No anaemia	24	2	26	26	0	26	-
Barkin Ladi (2009–2010)	Anaemia	8	4	12	8	2	10	
	No anaemia	34	6	40	34	4	38	0.59
Ibadan (2011–2014)	Anaemia	15	1	16	15	1	16	
	No anaemia	42	4	46	42	3	45	1.0
Total (2008–2014)	Anaemia	171	14	185	170	8	178	
	No anaemia	303	29	332	306	20	326	0.57

*PCR* polymerase chain reaction, *ACPR_u* adequate clinical and parasitological response uncorrected, *ACPR_c* adequate clinical and parasitological response corrected, *Failure_u* treatment failure uncorrected, *AA* artesunate-amodiaquine, *AL* artemether-lumefantrine, *ALL* all children

Overall, early treatment failure (ETF) occurred in 1 child treated with artesunate-amodiaquine. Late parasitological failure (LPF) occurred in 71 children: 37 of 818 children treated with artesunate-amodiaquine and 34 of 505 children treated with artemether-lumefantrine. There was no significant difference in the proportions of children with late parasitological failure in the two treatment groups (*P* = 0.11). There was also no significant difference in the proportions of children with late parasitological failure in anaemic and non-anaemic groups: 28 of 429 anaemic children *versus* 43 of 894 non-anaemic children (*P* = 0.24). The proportions of children with late parasitological failure did not increase over the years: 63 of 1 105 in 2008–2010 *versus* 8 of 218 in 2011–2014 (*P* = 0.12 test for trend).

##### Recrudescent and new infections

**Fig. 4:**
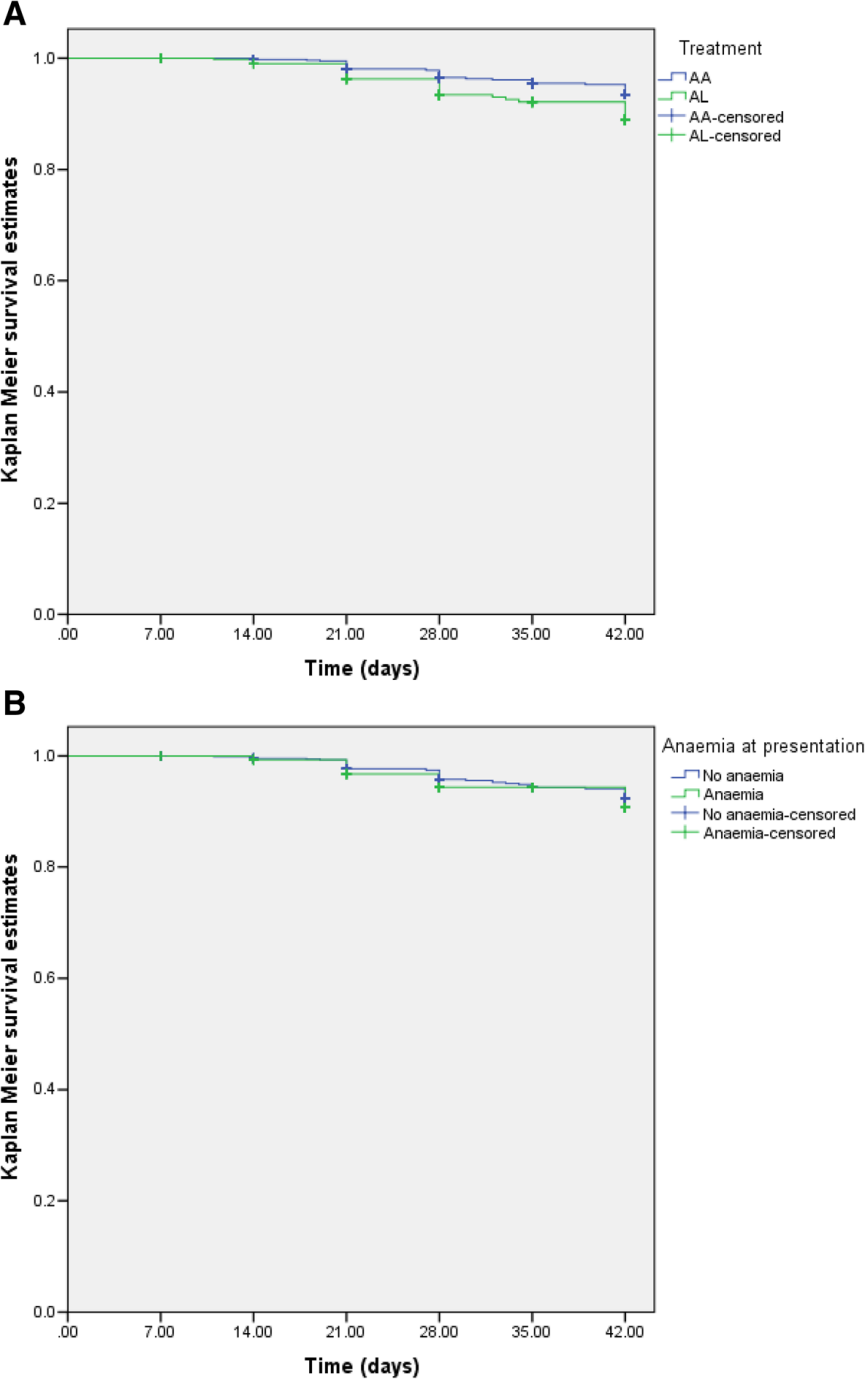
Kaplan-Meier survival estimates of asexual parasitaemia, (**a**) after treatment with AA (*blue line*) or AL (*green line*); [log-rank statistic = 7.37; *P* = 0.007], and (**b**) in children with (*green line*) or without (*blue line*) anaemia at presentation [log-rank statistic = 1.04; *P* = 0.31]. AA, artesunate-amodiaquine; AL, artemether-lumefantrine

Parasitaemia was detectable in 84 children before day 28–42. Of these, 17 were new infections, 57 were recrudescent infections of *P. falciparum* and in 10 cases, PCR results were inconclusive. Of the recrudescent infections, 27 were in children treated with artesunate-amodiaquine and 30 were in children treated with artemether-lumefantrine. The proportion of children with recrudescent infections was significantly higher in artemether-lumefantrine-treated group than in artesunate-amodiaquine-treated group (*P* = 0.03). However, there was no significant difference in the proportions of children with recrudescent infections in anaemic and non-anaemic children (16 of 437 *versus* 41 of 909, *P* = 0.56). Median time to recrudescent infections was similar in artesunate-amodiaquine- and artemether-lumefantrine - treated children (28 days (range 14–42) *versus* 28 days (range 11–42), *P* = 0.9). Similarly, time to recrudescent infections was similar in anaemic and non-anaemic children (28 days (range 14–42) *versus* 28 days (range 11–42), *P* = 0.94). Overall, the probabilities of reappearance of asexual parasitaemia after treatment were significantly higher with artemether-lumefantrine compared with artesunate-amodiaquine (Log-rank statistic = 7.37, *P* = 0.007, Fig. 4a). The probabilities of reappearance of asexual parasitaemia after treatment with the two (artesunate-amodiaquine or artemether-lumefantrine) were similar in anaemic and non-anaemic children (Log-rank statistic = 1.04, *P* = 0.31, Fig. 4b).

##### Prevalence of asexual parasitaemia on day 1

Overall, asexual parasite prevalence 1 day after treatment began was 34% (461 of 1 346 children). The prevalence was 28% (236 of 829 children) in children treated with artesunate-amodiaquine and 44% (225 of 517 children) in those treated with artemether-lumefantrine. The difference between these two proportions was significant (*P* < 0.0001). Parasite prevalence 1 day after treatment began was significantly higher in anaemic compared to non-anaemic children (175 of 437 children [40%] *versus* 286 of 909 children [31%]; *P* = 0.002).

##### Parasite positivity on day 3

Overall, parasite positivity on day 3 was 0.7% (9 of 1 346 children and it was similar in children treated with artesunate-amodiaquine or artemether-lumefantrine (6 of 829 *versus* 3 of 517 children, *P* = 1.0). Of the 286 children who had enrolment parasitaemia ≥100 000 μL^−1^, 3 children (2 in artesunate-amodiaquine and 1 in artemether-lumefantrine treatment groups) had parasitaemia on day 3, suggesting there was no in-vivo evidence of any cluster of cases with slow parasite clearance after treatment. Parasite positivity on day 3 was similar in anaemic and non-anaemic children (6 of 437 *versus* 3 of 909; *P* = 0.07). All 3 children with parasite positivity on day 3 who had parasitaemia ≥100 000 μL^−1^ at enrolment were anaemic at presentation.

##### Parasite reduction ratio 1 day after treatment began (PRR_D1_)

**Fig. 5:**
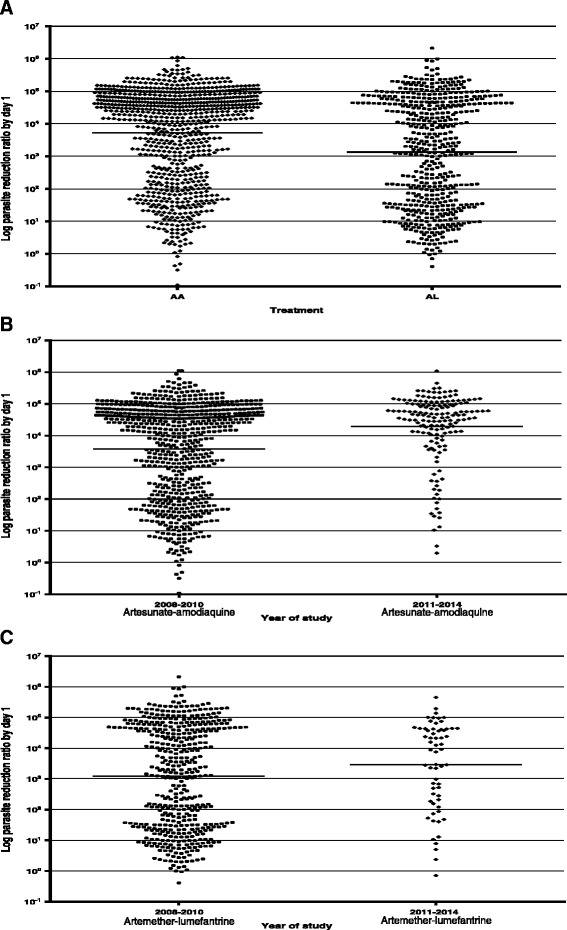
Scatter plots of day 1 parasite reduction ratios (PRR_D1_) in children with uncomplicated *P. falciparum* malaria following treatment with artesunate-amodiaquine (AA) or artemether-lumefantrine (AL): (**a**) all children treated with AA or AL during the period 2008–2014, (**b**) all children treated with AA during the period 2008–2010 and 2011–2014 and (**c**) all children treated with AL between 2008–2010 and 2011–2014

Overall, geometric mean parasite reduction ratio 1 day after treatment started was 3.1 × 10^3^ (range 1.1 × 10^−1^–2.1 × 10^6^). Geometric mean parasite reduction ratio 1 day after treatment began was significantly higher in artesunate-amodiaquine- compared to artemether-lumefantrine-treated children [5.2 × 10^3^ (range 1.1 × 10^−1^–1.1 × 10^6^) *versus* 1.3 × 10^3^ (range 4.1 × 10^−1^–2.1 × 10^6^); *P* < 0.0001] (Fig. 5a). PRR_D1_ was significantly higher in non-anaemic compared to anaemic children [4.3 × 10^3^ (range 3.2 × 10^−1^–1.1 × 10^6^) *versus* 1.6 × 10^3^ (range 1.1 × 10^−1^–2.1 × 10^6^); *P* < 0.0001]. PRR_D1_ increased with year following treatment and was significantly higher in 2011–2014 than in 2008–2010 with both ACTs [1.1 × 10^4^ (range 7.1 × 10^−1^–1.1 × 10^6^) *versus* 2.4 × 10^3^ (range 1.1 × 10^−1^–2.1 × 10^6^), *P* < 0.0001] (Fig. 5b and c).

##### Parasite reduction ratio 2 days after treatment began (PRR_D2_)

Overall, geometric mean parasite reduction ratio 2 days after treatment began was 2.5 × 10^4^ (range 1.6 × 10^1^–2.1 × 10^6^). Geometric mean parasite reduction ratio 2 days after treatment began was significantly higher in artesunate-amodiaquine- compared to artemether-lumefantrine-treated children [2.9 × 10^4^ (range 1.6 × 10^1^–1.1 × 10^6^) *versus* 2.0 × 10^4^ (range 1.7 × 10^1^–2.1 × 10^6^), *P* = 0.001]. PRR_D2_ was significantly higher in non-anaemic compared to anaemic children [2.9 × 10^4^ (range 1.7 × 10^1^–1.1 × 10^6^) *versus* 1.9 × 10^4^ (range 1.6 × 10^1^–2.1 × 10^6^); *P* < 0.0001]. PRR_D2_ increased over the years: it was significantly higher in 2011–2014 than in 2008–2010 with both ACTs [4.8 × 10^4^ (range 2.1 × 10^3^–1.1 × 10^6^) *versus* 2.2 × 10^4^ (range 1.6 × 10^1^–2.1 × 10^6^), *P* < 0.0001].

#### Secondary outcomes

##### Parasite and fever clearance

Overall, parasite clearance was significantly faster in artesunate-amodiaquine- compared with artemether-lumefantrine-treated children [1.3 day (95%*CI*: 1.3-1.4) *versus* 1.5 day (95%*CI*: 1.4-1.5), *P* < 0.0001]. Similarly, fever clearance was significantly faster in artesunate-amodiaquine- compared with artemether-lumefantrine-treated children [1.1 day (95%*CI*: 1.06 – 1.13) *versus* 1.2 day (95%*CI*: 1.13 – 1.29), *P* = 0.002]. The secondary outcomes in these children according to drug treatment or haematocrit status are shown in Table 3. Fever and parasite clearance times were similar in anaemic and non-anaemic children treated with artemether-lumefantrine. Fever but not parasite clearance time was similar in anaemic and non-anaemic children treated with artesunate-amodiaquine. Further exploratory analysis of the children treated with artesunate-amodiaquine when matched for age, gender, same treatment, same day of presentation and same parasitaemia, showed that, parasite and fever clearance times were similar in anaemic and non-anaemic children [1.2 day *versus* 1.1 day; *P* = 0.14 (*n* = 90) and 1.0 day *versus* 1.1 day; *P* = 0.18 (*n* = 90), respectively]. Parasite clearance time decreased significantly over the years [1.2 day (95%*CI*: 1.2-1.3) in 2011–2014 *versus* 1.4 day (95%*CI*: 1.4-1.5) in 2008–2010; *P* < 0.0001]. However, fever clearance did not change over the years [1.2 day (95%*CI*: 1.0-1.3) in 2011–2014 *versus* 1.1 day (95%*CI*: 1.1-1.2) in 2008–2010; *P* = 0.63].

**Table 3:** Fever and parasite clearance in malarious children following treatment with artesunate-amodiaquine or artemether-lumefantrine

	Artesunate-amodiaquine (829)		*P* value	Artemether-lumefantrine (517)		*P* value	All treatments (1 346)		*P* value
	No anaemia (577)	Anaemia (252)		No anaemia (332)	Anaemia (185)		No anaemia (909)	Anaemia (437)	
Fever clearance time (day)									
Mean 95%*CI*	1.1 1.0-1.1	1.1 1.1-1.2		1.2 1.1-1.2	1.3 1.1-1.5	0.09	1.1 1.08-1.14	1.2 1.11-1.28	0.03
Parasite clearance time (day)									
Mean 95%*CI*	1.3 1.2-1.3	1.4 1.3-1.5	0.01	1.5 1.4-1.5	1.5 1.4-1.6	0.21	1.4 1.3-1.4	1.4 1.4-1.5	0.14

##### Gametocyte carriage

Overall, 67 of 1 117 children (6%) had patent gametocytaemia at enrolment. Gametocyte carriage was similar in anaemic compared to non-anaemic children (19 of 295 *versus* 48 of 822, *P* = 0.71). In Ibadan where gametocyte carriage at presentation was evaluated for 7 years, gametocyte carriage did not decrease significantly over the study period (4 of 116, 15 of 255, 3 of 103, 7 of 68, 2 of 61 and 1 of 60 in 2008, 2009, 2010, 2011, 2012 and 2014, respectively, *P* = 0.41 test for trend). Gametocytes were not detectable in peripheral blood of all the children after day 14.

### Fall in haematocrit/1 000 asexual parasites cleared from peripheral blood

Data for evaluation of fall in haematocrit (FIH)/1 000 asexual parasites cleared from peripheral blood (cpb) were available in 643 children (see Fig. 3). Overall, median FIH/1 000 asexual parasites cpb was 0.029 (range 0.0001-0.91) and it did not decrease over the years [median 0.026 (range 0.0001-0.76; *n* = 443) in 2008–2010 *versus* 0.032 (range 0.0004–0.91, *n* = 200) in 2011–2014, *P* = 0.39]. FIH/1 000 asexual parasites cleared from peripheral blood was similar in children treated with artesunate-amodiaquine and artemether-lumefantrine [median 0.028 (range 0.0001–0.91, *n* = 441) *versus* 0.031 (range 0.0003–0.87, *n* = 202); *P* = 0.65]. FIH/1 000 asexual parasites cpb was significantly greater at lower parasitaemias (<100 000 μL^−1^) compared to higher parasitaemias (≥100 000 μL^−1^) [median 0.046 (range 0.001–0.91, *n* = 444) *versus* median 0.011 (range 0.0001–0.087, *n* = 199), *P* < 0.0001], suggesting much haematocrit conservation at higher parasitaemias compared to lower parasitaemias. In non-anaemic children, FIH/1 000 asexual parasites cleared from peripheral blood was significantly greater compared to anaemic children [median 0.032 (range 0.0001–0.91, *n* = 502) *versus* median 0.022 (range 0.0004–0.62, *n* = 141), *P* = 0.007] also suggesting much haematocrit conservation in anaemic compared to non-anaemic children. Similarly, FIH was significantly greater in patients with mild compared to moderate anaemia at presentation [median 0.024 (range 0.004–0.62, *n* = 133) *versus* median 0.002 (range 0.0008–0.09, *n* = 8), *P* = 0.04]. Further exploratory analysis showed that FIH/1 000 asexual parasites cleared from peripheral blood was significantly greater in non-anaemic children with enrolment parasitaemias of 50 000 μL^−1^ -100 000 μL^−1^ compared to anaemic children with enrolment parasitaemias of 50 000–100 000 μL^−1^ [median 0.037 (range 0.001–0.17, *n* = 126) *versus* median 0.026 (range 0.001–0.15, *n* = 48), *P* = 0.003].

### Anaemia recovery time

Anaemia recovery time was evaluated in patients who had ≥5 units fall in haematocrit from 30% and in whom haematocrit measurement was done consistently in >90% of the times of follow-up. Fifty eight of 185 anaemic children met the strict criteria for the evaluation of anaemia recovery time (see Fig. 3). Mean anaemia recovery time was 15.4 days (95%*CI:* 13.3–17.4). Anaemia recovery time did not change over the years (15.6 days (95%*CI:* 13–18.3, *n* = 39) in 2008 – 2010 *versus* 14.8 days (95%*CI:* 11.2–18.5, *n* = 19) in 2011–2014, *P* = 0.73). Anaemia recovery time was similar in children aged <3 and ≥3 years (18.5 days (95%*CI:* 13.7–23.4, *n* = 15) *versus* 14.3 days (95%*CI:* 12–16.5, *n* = 43); *P* = 0.07). Anaemia recovery time was also similar in anaemic children with high enrolment parasitaemias (≥100 000 μL^−1^) compared with those with low enrolment parasitaemias (<100 000 μL^−1^) [14.6 days (95%*CI:* 11.2–18, *n* = 20) *versus* 15.7 days (95%*CI:* 13–18.5, *n* = 38), *P* = 0.6]. There was no correlation between anaemia recovery time and parasite clearance time (*r* = 0.071, *P* = 0.58, *n* = 58) and between anaemia recovery time and FIH/1 000 asexual parasites cleared from peripheral blood (*r* = 0.004, *P* = 0.61, *n* = 58) in the same patients.

### Temporal changes in haematocrit in anaemic children following treatment with artesunate-amodiaquine or artemether-lumefantrine

Temporal changes in haematocrit were evaluated in 123 of 185 (66%) children who were anaemic at presentation and in whom haematocrit concentration was measured in all (100%) or nearly all (90%) of the follow-up period. The temporal changes in haematocrit are as follows: 1. Haematocrit <30% before treatment, followed by an increase to ≥30% after treatment and remaining so during the entire period of follow-up (malaria-associated anaemia and recovery from anaemia, *n* = 98 (79.7%)). 2. Haematocrit <30% at presentation followed by a rise to ≥30% by day 7 and a fall to <30% between days 7 and 14 and then recovery (anaemia-early recovery-early anaemia-late recovery, *n* = 12 (9.8%)). 3. Haematocrit <30% at presentation followed by a rise to ≥30% on day 7 or 14 and two consecutive normal haematocrit values followed by a decline to <30% after day 14 (anaemia-early recovery-late-appearing anaemia pattern, *n* = 7 (5.7%)). 4. Haematocrit <30% at before treatment began and during the entire follow-up period (persistent, unresolved anaemia, *n* = 4, (3.2%). 5. Patients with multiple falls below 30%, followed by multiple rises to ≥30% during follow-up period (undulating pattern of anaemia, *n* = 2 (1.6%)). No patient was unclassifiable.

The characteristics of the seven children with anaemia at presentation, who recovered from their anaemia and who subsequently developed late fall in haematocrit to <30% after day 14 are shown in Table 4. The late-appearing anaemia after initial recovery from the anaemia at presentation was characterised by an age ≤5 years (5 of 7 children), relatively high enrolment parasitaemia (6 of 7 had >50 000 asexual parasitaemia μL^−1^), rapid clearance of asexual parasitaemia [6 of 7 cleared by day 1] and low FIH/1 000 asexual parasites (cpb). Six of these 7 children were treated with artesunate-amodiaquine. Of the children treated with artesunate-amodiaquine, 4 were given total doses of artesunate ≥ 10 mg/kg over three days.

**Table 4:** Features of anaemic patients who recovered from their anaemia and had late fall in haematocrit below 30% (Pattern 3)^a^

Patient (gender, age)	Year of enrolment	Parasitaemia (μL^−1^)	Enrolment HCT (%)	Antimalarial treatment	PCT (day)	Nadir HCT^1^ (%) [day]	Nadir HCT^2^ (%) [day]	HCT on day 42 (%)	AnRT^1^ (day)	AnRT^2^ (day)	AUC^1^	AUC^2^	FIH/1 000 parasites cpb	Total of mg/kg dose of AA		Total of mg/kg dose of AL	
														Artesunate	Amodiaquine	Artemether	Lumefantrine
41 (F, 5y)	2011	315 429	28	AA	1	23 [3]	27[35]	33	14	7	25.82	6.17	0.0095	11.5	31.15	NA	NA
12 (M, 1.5y)	2014	98 327	25	AA	1	19 [3]	26[35]	28	14	NA	72.93	NA	0.0305	9.38	25.3	NA	NA
33 (M, 2.5y)	2010	70 823	28	AA	1	24 [3]	28[35]	28	14	NA	30.01	NA	0.0424	12.5	33.75	NA	NA
219 (F, 3y)	2008	31 640	23	AA	1	23 [0]	28[21]	31	14	7	49.83	4.67	0.0032	13.64	36.82	NA	NA
39 (M, 4.9y)	2010	183 741	29	AA	1	25 [2]	28[42]	28	7		12.44	NA	0.0054	8.82	23.82	NA	NA
15 (M, 1.2y)	2008	72 815	27	AL	1	26 [1]	28[28]	33	7	21	11.06	17.04	0.0137	NA	NA	17.4	102
20 (M, 5y)	2008	110 728	28	AA	2	17 [2]	28[35]	30	7	7	35.60	4.67	0.0542	10	27	NA	NA
Mean																	
Age (3.3y)		100 418^b^	26.86		1.14	22.43	27.6	30.14	11	10.5	33.95	8.14	0.023	10.98	29.64	17.4	102

*AA* artesunate-amodiaquine, *AL* artemether-lumefantrine, *HCT* Haematocrit, *PCT* Parasite clearance time, *AnRT* anaemia recovery time, *AUC* area under curve of deficit in haematocrit from 30% *versus* time, *FIH* fall in haematocrit, *cpb* cleared from peripheral blood, *NA* Not Applicable, *AUC* ^*1*^ AUC of anaemia at presentation, *AUC* ^*2*^ AUC of late fall in haematocrit; ^a^: many of these children were relatively asymptomatic when late-appearing anaemia occurred; ^b^: Geometric mean parasite density

No patient Reported symptoms during late-appearing anaemia

For comparison, the characteristics of 8 of 568 non-anaemic children at presentation who subsequently developed late-appearing anaemia after day 14 are shown in Table 5. The late-appearing anaemia was characterised by an age >5 years (7 of 8 children), relatively high enrolment parasitaemia (7 of 8 had parasitaemia >50 000 asexual parasitaemia μL^−1^), rapid clearance of asexual parasitaemia (5 of 8 cleared by day 1) and low FIH/1 000 asexual parasites cpb. Of these children, 6 were treated with artesunate-amodiaquine. Of the children treated with artesunate-amodiaquine, 5 were given total doses of artesunate ≥10 mg/kg over three days. Apart from age, all parameters appear to be similar in children with and without anaemia at presentation who developed late-appearing anaemia after day 14.

**Table 5:** Features of non-anaemic patients at presentation who subsequently developed late fall in haematocrit below 30% 3 – 6 weeks later^a^

Patient (gender, age)	Year of enrolment	Parasitaemia (μL^−1^)	Enrolment HCT (%)	Antimalarial treatment	PCT (day)	Nadir HCT^2^ (%) [day]	HCT on day 42 (%)	AnRT^1^ (day)	AnRT^2^ (day)	AUC^2^	FIH/1 000 parasites cpb	Total of mg/kg dose of AA		Total of mg/kg dose of AL	
												Artesunate	Amodiaquine	Artemether	Lumefantrine
13 (F, 4.9y)	2010	357 397	40	AA	2	27[21]	30		14	24.68	0.0113	11.54	31.15	NA	NA
39 (M, 9y)	2012	2 880	37	AL	1	24[21]	32		7	6.57	0.3472	NA	NA	12.63	75.79
62 (M, 12y)	2010	50 704	35	AA	2	27[42]	27		NA	NA	0.0592	8.57	23.14	NA	NA
153 (F, 8y)	2009	101 734	35	AA	1	28[28]	32		7	2.64	0.0295	15	40.5	NA	NA
71 (M, 6y)	2009	178 937	35	AL	1	28[21]	34		7	2.64	0.0112	NA	NA	16.1	96.0
129 (M, 9y)	2008	99 077	33	AA	1	29[21]	45		7	1.52	0.0202	13.04	35.22	NA	NA
61 (M, 8.1y)	2014	77 727	33	AA	1	20[28]	37		7	10.13	0.0386	15.79	42.63	NA	NA
40 (F, 5.3y)	2011	281 538	32	AA	2	29(35)	34		7	1.52	0.0074	10.00	27.0	NA	NA
Mean															
Age (7.8y)		82 078^b^	35		1.38	27.6	33.88		8	7.1	0.066	12.32	33.27	14.36	85.9

*AA* artesunate-amodiaquine, *AL* artemether-lumefantrine, *HCT* Haematocrit, *PCT* Parasite clearance time, *AnRT* anaemia recovery time, *AUC* area under curve of deficit in haematocrit from 30% *versus* time, *FIH* fall in haematocrit, *cpb* cleared from peripheral blood, *NA* Not Applicable, *AUC* ^*2*^ AUC of late fall in haematocrit; ^a^: many of these children were relatively asymptomatic when late-appearing anaemia occurred; ^b^: Geometric mean parasite density

No patient Reported symptoms during late-appearing anaemia

### Relationship between AUC of deficit in haematocrit from 30% *versus* time and FIH/1 000 asexual parasites cleared from peripheral blood

In the 7 children who were anaemic at presentation, who recovered from their anaemia and who subsequently developed anaemia 21 or more days after treatment began (see Table 4), the AUC of deficit in haematocrit from 30% *versus* time at presentation were similar to AUC of deficit in haematocrit from 30% *versus* time when anaemia developed 21 or more days after treatment began [33.9%.day (95%*CI*: 13.83–54.1) *versus* 19.9%.day (95%*CI*: 3.62–48.36); *P* = 0.09]. There was no correlation between AUC of deficit in haematocrit from 30% *versus* time of the late-appearing anaemia and FIH/1 000 asexual parasites cleared from peripheral blood (*r* = 0.45; *P* = 0.31).

### Kinetics of the disposition of the deficit in haematocrit from 30%

**Fig. 6:**
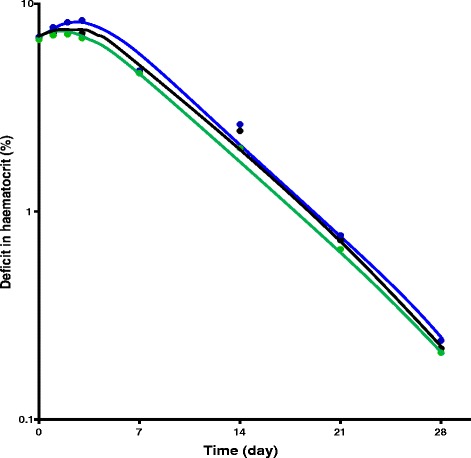
Semilog plots of deficit in haematocrit from 30% *versus* time in all children treated with artesunate-amodiaquine or artemether-lumefantrine (*black line*) and in children treated with artesunate-amodiaquine (*green line*) or artemether-lumefantrine (*blue line*)

The kinetics of the disposition of the deficit in haematocrit were evaluated in 58 children (*n* = 43 for artesunate-amodiaquine and *n* = 15 for artemether-lumefantrine) with the following demographic and other characteristics: mean age 5.2 years (range 1.1-13); mean duration of illness 3.5 days (range 1–7); mean body temperature 38.4°C (range 36.2-41); mean haematocrit 23.2% (range 17–25); geometric mean parasitaemia 61 752 asexual form /μL (range 2 100 – 288 461); mean fever clearance time 1.2 days (1–7); mean parasite clearance time 1.2 days (range 1–3); mean anaemia recovery time 15.4 days (range 2–28). None of the patients had late-appearing anaemia. Overall, there was monoexponential decline of the deficit in haematocrit from 30% with an estimated mean elimination half-time (t_½el_, t_½anaemia_) of 1.4 days (95%*CI*: 1.2-1.6) (Fig. 6). In <5 (*n* = 34) and ≥5 (*n* = 24) year olds, estimated mean elimination half-times were 1.5 days (95%*CI*: 1.2-1.8) and 1.3 days (95%*CI*: 0.9-1.6) and they were similar (*P* = 0.38). The estimated mean t_½el_ values were also similar in artesunate-amodiaquine- (1.4 days, 95%*CI*: 1.1–1.7) and artemether-lumefantrine-treated (1.3 days, 95%*CI*: 0.9–1.7) children (*P* = 0.66) (Fig. 6). Estimated mean half-times were also similar in children with enrolment parasitaemia ≥100 000 μL^−1^ and those with <100 000 μL^−1^ [1.2 days (95%*CI*: 0.9–1.4, *n* = 20) *versus* 1.5 days (95%*CI*: 1.2–1.8, *n* = 38); *P* = 0.19], and in children with mild and moderate anaemia at presentation [1.4 days (95%*CI*: 1.1–1.6, *n* = 48) *versus* 1.6 days (95%*CI*: 1.1–2.0, *n* = 10); *P* = 0.46].

Overall, mean areas under curve of deficit in haematocrit from 30% *versus* time (AUC_def_) value were 63.7%.day (95%*CI*: 51.0–76.4). Mean AUCs were similar in artesunate-amodiaquine- and artemether-lumefantrine - treated children [59.4%.day (95%*CI*: 46.3–72.5) *versus* 76.0%.day (95%*CI*: 41.7–110.3), *P* = 0.26]. Mean AUC_def_ values were also similar in <5 and ≥5 year olds [65.2%.day (95%*CI*: 49.9–80.4, *n* = 34) and 61.6%.day (95%*CI*: 38.3–84.8, *n* = 24), *P* = 0.78], and in children with enrolment parasitaemia ≥100 000 μL^−1^ and those with <100 000 μL^−1^ [65.8%.day (95%*CI*: 43.8–87.8; *n* = 20) and 62.5%.day (95%*CI*: 46.3–78.8; *n* = 38), *P* = 0.81].

### Relationship between half - time of decline in haematocrit deficit and anaemia recovery time

**Fig. 7:**
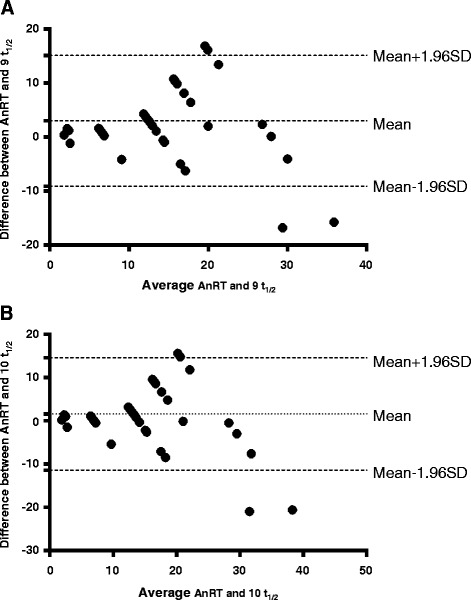
Bland-Altman plots of anaemia recovery times and multiples [9 (**a**) and 10 (**b**)] of anaemia half-times. Biases were 3.0, and 1.6 for plots A and B; *P* = 0.0005 and 0.07; respectively. The mean values ± 1.96 standard deviation (SD) of the differences are shown. AnRT; Anaemia recovery time, t_½_; half-time

The relationship between the half-time of decline in haematocrit deficit from 30% and anaemia recovery time in the same patients with anaemia at presentation was evaluated in 58 children. The mean half-time of decline in haematocrit deficit from 30% was 1.4 days (95%*CI*: 1.2–1.6). The mean anaemia recovery time was 15.4 days (range 2–28). There was a significantly positive correlation between half-time of decline in haematocrit deficit from 30% and anaemia recovery time in the same patients (*r* = 0.69, *P* < 0.0001). Bland-Altman plots of the anaemia recovery times and 9 or 10 multiples of anaemia half-times are shown in Fig. 7. The limit of agreement between anaemia recovery time and 9 multiples of anaemia half-time was not narrow. The bias was significantly different from 0 (*P* = 0.0005). However, at multiple of 10 half-times, the limit of agreement between anaemia half-time and anaemia recovery time was narrow. The bias at multiple of 10 anaemia half-times was statistically insignificant (*P* = 0.07).

### Adverse events

Adverse events were carefully monitored in 610 non-anaemic and 185 anaemic children drawn from Ibadan centre. Overall, 186 of 795 children [23%] (150 of 548 [27%] in artesunate-amodiaquine and 36 of 247 [15%] in artemether-lumefantrine reported at least one adverse event within the first week of starting treatment. There was a significant difference in the proportions of children reporting adverse events in both treatment groups (*P* < 0.0001). Fever (70 of 548 [13%] *versus* 11 of 247 [4%], *P* = 0.0005), vomiting (38 of 548 (7%) *versus* 6 of 247 (2%), *P* = 0.016), headache (33 of 548 (6%) *versus* 4 of 247 (2%), *P* = 0.006), abdominal pain (46 of 548 (8%) *versus* 3 of 247 (1%), *P* = 0.00002) and anorexia (26 of 548 (5%) *versus* 3 of 247 (1%), *P* = 0.013) were significantly more common in artesunate-amodiaquine- compared with artemether-lumefantrine-treated children. Other reported adverse events (cough, weakness, puffy face, itching and drowsiness) were similar in frequency in the two treatment groups. One hundred and thirty eight of 610 children without anaemia at presentation [23%] and 48 of 185 children with anaemia at presentation [26%] reported at least one adverse event within the first week of commencement of treatment. There was no significant difference in the proportions of children reporting adverse events in anaemic and non-anaemic children (*P* = 0.4). The most commonly reported adverse events in non-anaemic and anaemic children were fever [65 of 610 (11%) *versus* 16 of 185 (9%), *P* = 0.62], vomiting [35 of 610 (6%) *versus* 9 of 185 (5%), *P* = 0.21], abdominal pain [31 of 610 (5%) *versus* 18 of 185 (10%), *P* = 0.03] and cough [40 of 610 (7%) *versus* 19 of 185 (9%), *P* = 0.09].

## Discussion

In this study, conducted during a seven year-period of adoption of ACTs as first-line treatments of uncomplicated *P. falciparum* malaria in Nigeria, artesunate-amodiaquine proved a superior alternative to artemether-lumefantrine as evidenced by a significantly higher PCR-corrected 28 days parasitological efficacy or better measures of therapeutic responses. These findings were not unexpected as a previous relatively large study conducted in southwest Nigeria during the first 5 years of adoption showed similar results [36]. The PCR-corrected 28 days efficacy of over 96% with both treatments supports continuing efficacy of ACTs in *P. falciparum* infections in virtually all endemic areas of Nigeria since adoption as first-line treatments in 2005. It is, however, in contradistinction to the reports of declining responsiveness of *P. falciparum* to ACTs in Kenya or Suriname [53, 54] or resistance to artemisinin in the Greater Mekong subregion [5, 7].

It is intriguing that PRR_D1_, a less frequently evaluated measure of therapeutic efficacy in the area of study, was also significantly higher in children treated with artesunate-amodiaquine compared with artemether-lumefantrine. With respect to parasite prevalence 1 day after treatment began being significantly lower in children treated with artesunate-amodiaquine compared to those treated with artemether-lumefantrine, similar difference in parasite prevalence after treatment began has been reported between the two ACTs [55].

Overall, despite being significantly younger, parasitological responses in anaemic children were similar to those of non-anaemic children suggesting that in this area of full sensitivity in *P. falciparum* to the two ACTs, anaemia did not compromise, to any significant extent, the therapeutic responses to both treatments. The younger age of the anaemic children may be partly responsible for the slower parasite clearance and the lower parasite reduction ratios 1 and 2 days after treatment began in artesunate-amodiaquine-treated children because younger children may be considered to have relatively lower antimalarial immunity [56] and therefore slower response.

The present study showed that ACTs conserved haematocrit significantly at high parasitaemias compared to low parasitaemias, in anaemic compared to non-anaemic children, and in children with moderate compared to mild anaemia. The reasons for haematocrit conservation are unclear. It is possible the mechanisms and the kinetics of the production and disposition of the once-infected red blood cells may differ in anaemic and non-anaemic children in the first few or more days after start of artemisinin-based combination treatments. In this context, studies are needed on the production and disposition kinetics of infected and once-infected red blood cells in anaemic and non-anaemic children in this endemic area following artemisinin-based combination treatments.

Successful treatment of *P. falciparum* malaria with ACTs is often followed by increases in haematocrit or haemoglobin. This often led to recovery from uncomplicated malaria-associated anaemia [13, 17, 35, 40, 57]. In this relatively large series of anaemic children, anaemia recovery time in children with ≥5 units deficit in haematocrit from 30% was approximately 2 weeks. This recovery time is similar to that recently reported in very young children [58].

The monoexponential declines of the deficits in haematocrit from 30% would suggest that, using a non-compartment model, recovery from uncomplicated malaria-associated anaemia is a first-order process [47]. The anaemia recovery time : anaemia half-time of 10, and the insignificant bias between anaemia recovery time and 10 multiples of anaemia half-time by Bland-Altman analysis suggest that anaemia recovery time and 10 multiples of anaemia half-time can be used interchangeably in the evaluation of recovery from uncomplicated *P. falciparum* malaria-associated anaemia. This finding was expected because in a simple one-compartment pharmacokinetic model approximately 99.9% of an elimination process would have been completed in 10 half-times [48]. Thus, there is a pharmacokinetic equivalent (anaemia half-time) of a pharmacodynamic process (anaemia recovery time) in the same patients.

In children who were anaemic at presentation, the commonest temporal change in haematocrit following treatment was recovery from the associated anaemia. However, a relatively asymptomatic late-appearing anaemia occurred in 6% of anaemic children, who initially recovered from their malaria-associated anaemia following treatment. It would also appear late-appearing anaemia was significantly more frequent in anaemic compared to non-anaemic children. The relative absence of symptoms when late-appearing anaemia occurred, may make it difficult to diagnose late-appearing anaemia in children with uncomplicated infections following artemisinin-based combination treatments. The absence of overt symptoms and signs of acute haemolysis and uneventful recovery from the late-appearing anaemia would suggest that late-appearing anaemia is a previously unrecognised feature of artemisinin-based combination treatments in African children with uncomplicated infections. Studies are now under way in this endemic area of Nigeria to evaluate the risk factors associated with the relatively asymptomatic late-appearing anaemia after artemisinin-based combination treatments of uncomplicated *P. falciparum* malaria in children.

In general, the reported adverse events within the first week of starting treatment were indistinguishable from the symptoms of malaria. The significantly higher frequency of reported adverse events in those treated with artesunate-amodiaquine compared with artemether-lumefantrine are in keeping with previous report [59]. Pruritus has been reported in Nigeria children treated with artemether-lumefantrine [60] but in this relatively large series no child reported pruritus following artemether-lumefantrine treatment.

There are limitations of the present studies. First, although the clinical and parasitological features of children with fall in haematocrit below 30% at presentation were characterised, the nature of the anaemia was not fully characterized (that is, whether it was haemolytic or not in nature). Second, in the children with late fall in haematocrit below 30%, quantification of once-infected and infected red blood cells and the disposition of these red blood cells during the course of follow-up were not done. Third, in children with anaemia before or following treatment with ACTs, the contribution of background causes of anaemia in the area of study namely nutritional, helminthic infections, or glucose-6-phosphate dehydrogenase deficiency was not evaluated.

## Conclusion

In conclusion, artesunate-amodiaquine and artemether-lumefantrine remain efficacious treatments of uncomplicated *P. falciparum* infections in non-anaemic and anaemic Nigerian children in the last 7 years of adoption as first-line treatments. These ACTs may also conserve haematocrit at high parasitaemias and in anaemic children.

## Additional file

Additional file 1: Multilingual abstracts in the six official working languages of the United Nations. (PDF 808 kb)

## Abbreviations

%: Percent
°C: Degree celsius
AA: Artesunate-amodiaquine
ACPR: Adequate clinical and parasitological response
ACTs: Artemisinin-based combination treatments
AL: Artemether-lumefantrine
ANOVA: Analysis of variance
AnRT: Anaemia recovery time
AUC: Area under curve
d: Day
ETF: Early treatment failure
FCT: Fever clearance time
FIH: Fall in haematocrit
g: Gram
HCT: haematocrit
kg: Kilogram
L: Litre
LPF: Late parasitological failure
M/F: Male/female
mg: Milligram
PCR: Polymerase chain reaction
PCT: Parasite clearance time
PRR: Parasite reduction ratio
PRR_D1_: Parasite reduction ratio on day 1
PRR_D2_: Parasite reduction ratio on day 2
sd: Standard deviation
t_½_: Half-time
μL: Microliter
