# Supplementary material for: Therapeutic efficacy and effects of artemisinin-based combination treatments on uncomplicated Plasmodium falciparum malaria -associated anaemia in Nigerian children during seven years of adoption as first-line treatments
Source: Infect Dis Poverty. 2017 Feb 7;6:36. doi: 10.1186/s40249-016-0217-7 (PMC5294876; doi:10.1186/s40249-016-0217-7)
Supplement: Additional file 1: — Multilingual abstracts in the six official working languages of the United Nations. (PDF 808 kb) [file 40249_2016_217_MOESM1_ESM.pdf]

Translation of the abstract into the six official working languages of the United Nations

الكفاءة العلاجية وآثار العلاجات التوليفية القائمة على مادة الأرتيميسينين على الأنيميا غير المعقدة المصاحبة للمتصورة المنجلية في الأطفال النيجيريين خلال سبع سنوات من اعتمادها الخيار الأول في العلاج

اكينتوندي سووومني، كاظيم أكانو، جودوين نتادوم، أديجوموكي أولا أبيدي، فولاسادي و. إيبيرونكي، تيميتوبي أديروييجي، إلسي و. أديوويي، بايو فاتونمبي، ستيفن أوجوتشي، هنريتا و. أوكافور، إسماعيل واتيلا، مارتن ميريميكوو، فيليب أجومو، وليام أوجالا، تشيميري أوجومو، أونيكيبي أ. فولارين، جريس و. جبوتوشو، كريستيان هابي

ملخص

خلفية: -العلاجات التوليفية القائمة على على الأرتيميسينين (ACTs) هي الخيار الأول في العلاج من ملاريا المتصورة المنجلية غير المعقدة في العديد من المناطق الموبوءة ولكن هناك عدد قليل من التقييمات لفعاليتها في الأطفال المصابين بالأنيميا المصحوبة بالملاريا.

الأساليب: الفاعلية العلاجية لعلاج من 3 أيام من الأرتيسونات-أمودياكين وأرتيميثير-اللوميفانترين تم تقييمها في حالات طفلا مصابا بالملاريا 437 مصحوبة بأنيميا و 909 غير مصحوبة بأنيميا بعد العلاج خلال فترة سبع سنوات (2008-2014). تم تصنيف أنماط التغيرات الزمانية في الهيماتوكريت على أساس قيم الهيماتوكريت <30 %و≤30%. تم تقييم حركية التخلص من العجز في الهيماتوكريت من 30% بعد العلاج باستخدام نموذج من دائرة واحدة.

النتائج: تحليل الفعالية الطفيلية لتفاعل البوليميراز المتسلسل لمدة 28 يوما بعد بدء العلاج بدا أعلى بكثير في الأرتيسونات-أموياكوين مقارنة بالأطفال المعالجين بأرتيميثير- اللوميفانترين [97% (95% CI 92.8-100) مقابل 96.4% (95% CI91.3-99.4)، P = 0.02]، ولكنه كان مماثلا في الأطفال غير المصابين بالأنيميا والأنيميا. كان الانخفاض في الهيماتوكريت /1000من الطفيليات اللاجنسية التي تم تنظيفها من الدم المحيطي أكبر بكثير في عدد الطفيليات الأقل في كريات الدم الحمراء مقارنة بالأعلى (P <0.0001)، وفي غير المصابين بأنيميا مقارنة مع الأطفال المصابين بالأنيميا (P=0.007). في الأطفال المصابين بالأنيميا ، كان وقت التعافي من الأنيميا (ANRT) 15.4 يوما (95% CI 13.3-17.4)، ولم يتغير خلال فترة العلاج. كانت الانخفاضات في عجز الهيماتوكريت 30% أحادي متخامد مع متوسط وقت النصف المقدر بـ1.4 يوما (95% CI1.2-1.6). تناسب وقت النصف للأنيميا (t½anaemia) طرديا مع ANRT في نفس المرضى (r= 0.69، *P*<0.0001). وأظهر تحليل بلاند-ألتمان لـ10 مضاعفات t½anaemia وANRT قدرا ضئيلا من الانحياز يمكن تجاهله (*P*=0.07) مما يدل على إمكانية استخدام الاثنين على حد سواء بالتبادل في نفس المرضى.

الاستنتاجات: تبقى الارتيسونيت-أمودياكين وأرتيميثير-اللوميفانترين العلاجات الفعالة لالتهابات المتصورة المنجلية غير المعقدة في الأطفال النيجيريين غير المصحوبة بأنيميا والمصحوبة بأنيميا في آخر 7 سنوات من اعتمادها الخيار الأول في العلاج. قد يحافظ الأرتيميسينين أيضا على الهيماتوكريت عند ارتفاع عدد الطفيليات في كريات الدم الحمراء وفي الأطفال المصابين بالأنيميا.

تسجيل التجارب: تسجيل التجارب السريرية في عموم أفريقيا PACTR201508001188143، 3 يوليو 2015؛ PACTR20151000118937، 3 يوليو 2015؛ PACTR201508001191898، 7 يوليو عام 2015، PACTR201508001193368، 8 يوليو 2015.

Translated from English version into Arabic by Mahmoud Sami, through

TRANSLATORS
WITHOUT BORDERS

7年间对非重症恶性疟相关性贫血尼日利亚儿童以青蒿素为基础的联合治疗为一线疗法的疗效和效应

Akintunde Sowunmi, Kazeem Akano, Godwin Ntadom, Adejumoke I. Ayede, Folasade O. Ibironke, Temitope Aderoyeje, Elsie O. Adewoye, Bayo Fatunmbi, Stephen Oguche, Henrietta U. Okafor,

Ismaila Watila, Martin Meremikwu, Philip Agomo, William Ogala, Chimere Agomo, Onikepe A. Folarin, Grace O. Gbotosho, Christian Happi

摘要

引言：以青蒿素为基础的联合治疗方法在很多疟疾流行地区都作为针对非重症恶性疟的一线疗法，但是该疗法对疟疾性贫血儿童的疗效尚缺乏评估。

方法：2008 年到 2014 年 7 年间，对 437 例贫血和 909 例非贫血的疟疾儿童中评估青蒿琥酯-阿莫地喹和青蒿琥酯-苯芴醇 3 天用药方案的疗效。根据血细胞比容值<30%和≥30%对血细胞比容时间变化模式进行分类，采用单组份模型评估 30%后续治疗血细胞比容不足的动力学。

结果：治疗后 28 d，青蒿琥酯-阿莫地喹治疗组 PCR 校正原虫疗效[97% (95% *CI*: 92.8–100)]高于青蒿琥酯-苯芴醇治疗组[96.4% (95% *CI*: 91.3–99.4), *P*=0.02]，但是对贫血儿童和非贫血儿童疗效相似。与高原虫血症比，低原虫血症患者血细胞比容/1 000 无性体原虫在外周血清除率明显高于高原虫血症者(*P*<0.0001)，非贫血患儿的清除率高于贫血患儿(*P*=0.007)。在贫血儿童中，平均贫血恢复时间 15.4 d (95% *CI*: 13.3–17.4)，且不随着年份而变化。血细胞比容从 30%下降至不足与平均估计半衰期 1.4 d (95% *CI*: 1.2–1.6)间为单一指数关系。在同一患者中，贫血半衰期($t_{½anaemia}$)与平均贫血恢复时间（AnRT）正相关(*r*=0.69, *P*<0.0001) 。Bland-Altman 分析 10 倍 $t_{½anaemia}$ 和 AnRT 表现出有限的一致性并且存在非显著性偏倚(*P*=0.07)，这表明这两项指标可以在同一患者中交互使用。

结论：在过去 7 年间，青蒿琥酯-阿莫地喹和青蒿琥酯-苯芴醇作为一线疗法在尼日利亚治疗贫血和非贫血疟疾患儿的治疗中仍然是有效的。这类 ACTs 药物能够在高原虫血症和贫血儿童中维持血细胞比容。

Translated from English version into Chinese by Xin-Yu Feng, edited by Pin Yang

CDC CHINESE CENTER FOR DISEASE CONTROL AND PREVENTION
NATIONAL INSTITUTE OF PARASITIC DISEASES

Efficacité thérapeutique et effets des traitements combinés à base d'artémisine sur l'anémie non complexe associée au *Plasmodium falciparum* chez des enfants nigérians pendant une période de sept ans d'adoption de traitements de première ligne

Akintunde Sowunmi, Kazeem Akano, Godwin Ntadom, Adejumoke I. Ayede, Folasade O. Ibironke, Temitope Aderoyeje, Elsie O. Adewoye, Bayo Fatunmbi, Stephen Oguche, Henrietta U. Okafor, Ismaila Watila, Martin Meremikwu, Philip Agomo, William Ogala, Chimere Agomo, Onikepe A. Folarin, Grace O. Gbotosho, Christian Happi

Résumé

Contexte: les traitements combinés à base d'artémisine (ACT) constituent les traitements de première ligne du paludisme à *Plasmodium falciparum* non complexe dans de nombreuses zones endémiques, mais très peu d'évaluations de leur efficacité ont été réalisées chez des enfants anémiques atteints du paludisme.

Méthodes: l'efficacité thérapeutique de schémas thérapeutiques de 3 jours à base d'artésunate-amodiaquine et d'artéméther-luméfrantine a été évaluée à la suite d'un traitement auprès de 437 enfants anémiques et de 909 enfants non anémiques, tous atteints de paludisme, pendant une

durée de sept ans (2008-2014). Les modèles de changement temporel de l'hématocrite ont été classés en fonction des valeurs de l'hématocrite <30% et ⩾30%. La cinétique de la disposition de la diminution de l'hématocrite à partir de 30% à la suite d'un traitement a été évaluée à l'aide d'un modèle non compartimental.

Résultats: l'efficacité parasitologique corrigée par PCR 28 jours après le lancement du traitement était nettement plus élevée chez les enfants traités par l'artésunate-amodiaquine par rapport aux enfants traités par l'artéméther-luméfantrine (97% [*IC* à 95%: 92,8–100] *versus* 96,4% (*IC* à 95%: 91,3–99,4), *P*=0,02), mais elle était similaire chez les enfants non anémiques et anémiques. La réduction de l'hématocrite/1000 parasites asexuels éliminés du sang périphérique était nettement plus importante en cas de faible parasitémie par rapport à une parasitémie élevée (*P*<0,0001) et chez les enfants non anémiques par rapport aux enfants anémiques (*P*=0,007). Au moment de leur présentation, les enfants anémiques présentaient une durée moyenne de récupération de l'anémie (AnRT) de 15,4 jours (*IC* à 95%: 13,3–17,4) et cette dernière n'a pas évolué au cours des années. Les diminutions des déficits d'hématocrite à partir de de 30 % étaient monoexponentielles et affichaient une demi-vie estimée de 1,4 jour (*IC* à 95%: 1,2–1,6). L'anémie de demi-vie ($t_{½\text{anémie}}$) présentait une corrélation positive avec l'AnRT chez les mêmes patients (*r*=0,69, *P*<0,0001). L'analyse de Bland-Altman de 10 multiples de la $t_{½\text{anémie}}$ et de l'AnRT a démontré une étroite limite d'accord associée à un biais minime (*P*=0,07) suggérant l'interchangeabilité des deux traitements chez les mêmes patients.

Conclusions: l'artésunate-amodiaquine et l'artéméther-luméfantrine restent des traitements efficaces des infections à *P. falciparum* non complexes chez les enfants nigérians non anémiques et anémiques au cours des sept dernières années d'adoption à titre de traitements de première ligne. Ces CTA peuvent aussi maintenir l'hématocrite à des niveaux de parasitémie élevés et chez des enfants anémiques.

Enregistrement d'essais cliniques: Registre panafricain des essais cliniques PACTR201508001188143, 3 juillet 2015; PACTR201510001189370, 3 juillet 2015; PACTR201508001191898, 7 juillet 2015 et PACTR201508001193368, 8 juillet 2015.

Translated from English version into French by eric ragu, through

TRANSLATORS WITHOUT BORDERS

Оценка терапевтической эффективности и эффекта комплексного лечения на основе артемизинина неосложненной анемии у нигерийских детей, вызванной паразитом *Plasmodium falciparum,* на протяжении семи лет применения препарата в качестве терапии первого ряда

Акинтунде Совунми, Казим Акано, Гудвин Нтадом, Адеймук И.Айед, Фоласад О.Ибиронке, Темитоп Адеройей, Элзи О.Адевойе, Байо Фатунмби, Стивен Огуче, Генриетта У.Окафор, Исмаила Ватила, Мартин Меремикву, Филипп Агомо, Уильям Огала, Химер Агомо, Оникепе А.Фоларин, Грейс О.Гботошо, Кристиан Хаппи (Akintunde Sowunmi, Kazeem Akano, Godwin Ntadom, Adejumoke I. Ayede, Folasade O. Ibironke, Temitope Aderoyeje, Elsie O. Adewoye, Bayo

Fatunmbi, Stephen Oguche, Henrietta U. Okafor, Ismaila Watila, Martin Meremikwu, Philip Agomo, William Ogala, Chimere Agomo, Onikepe A. Folarin, Grace O. Gbotosho, Christian Happi)

Отрывок

История вопроса: Комплексное лечение на основе артемизинина применяется в качестве терапии первого ряда при лечении неосложненной малярии, вызванной паразитом *Plasmodium falciparum,* во многих эндемичных районах, однако оценка ее эффективности при лечении анемичных детей, больных малярией, проводилась редко.

Методы: Была проведена оценка терапевтической эффективности 3-дневной схемы лечения артесунат-амодиахином и артеметер-люмефантрином среди 437 анемичных и 909 неанемичных детей, больных малярией, в результате семилетнего лечения (2008-2014 гг.). Модели изменений гематокрита во времени были классифицированы на основании значений гематокрита <30% и $\geqslant$30%. Динамика предрасположенности к недостатку гематокрита с 30% по результатам лечения оценивалась посредством некомпартментной модели.

Результаты: Скорректированная на основании ПЦР паразитологическая эффективность через 28 дней после начала терапии была значительно выше у детей, лечение которых проводилось артесунат-амодиахином, чем у детей, лечение которых проводилось артеметер-люмефантрином [97% (95%*CI* 92,8–100) *по сравнению с* 96,4% (95%*CI* 91,3–99,4), *P*=0,02], но этот показатель был равным среди анемичных и неанемичных детей. Показатель снижения гематокрита/1 000 бесполых паразитов, выведенных из периферической крови, был значительно ниже по сравнению с более высоким уровнем паразитемии (*P*<0,0001), и среди неанемичных детей, в сравнении с анемичными детьми (*P*=0,007). При первичном обследовании анемичных детей, среднее значение периода восстановления после анемии (AnRT) составляло 15,4 дня (95%*CI* 13,3–17,4), и оно не менялось на протяжении лет. Снижение уровня дефицита гематокрита с 30% было моноэспоненциальным, со средним полупериодом в 1,4 дня (95%*CI* 1,2–1,6). Среди этих же пациентов была отмечена положительная зависимость между периодом полувыведения при анемии ($t_{½anaemia}$) и показателем AnRT (*r*=0,69, P<0,0001). Анализ Бланда-Альтмана 10 кратных показателей $t_{½anaemia}$ и AnRT продемонстрировал ограниченный предел соответствия, с незначительным отклонением (*P*=0,07), из чего можно сделать вывод, что оба метода лечения могут равноценно применяться у одних и тех же пациентов.

Заключение: Терапия артесунат-амодиахином и терапия артеметер-люмефантрином остаются эффективными методами лечения неосложненных инфекций, вызванных паразитом *P. falciparum,* как анемичных, так и неанемичных нигерийских детей за последние 7 лет их применения в качестве терапии первого ряда. Данные виды комплексного лечения на основе артемизинина также могут способствовать сохранению уровня гематокрита при высоком уровне паразитемии у анемичных детей.

Регистрация испытаний: Панафриканский реестр клинических испытаний PACTR201508001188143, 3 июля 2015 г.; PACTR201510001189370, 3 июля 2015 г.; PACTR201508001191898, 7 июля 2015 г. и PACTR201508001193368, 8 июля 2015 г.

Translated from English version into Russian by tatiana_com, through

TRANSLATORS
WITHOUT BORDERS

Eficacia terapéutica y los efectos de los tratamientos combinados con artemisinina en anemia sin complicaciones asociadas con *Plasmodium falciparum*en niños nigerianos durante los siete años siguientes a la adopción como tratamiento de primera línea

Akintunde Sowunmi, Kazeem Akano, Godwin Ntadom, Adejumoke I. Ayede, Folasade O. Ibironke, Temitope Aderoyeje, Elsie O. Adewoye, Bayo Fatunmbi, Stephen Oguche, Henrietta U. Okafor, Ismaila Watila, Martin Meremikwu, Philip Agomo, William Ogala, Chimere Agomo, Onikepe A. Folarin, Grace O. Gbotosho, Christian Happi

Resumen

Historia: Los tratamientos combinados basados en la artemisinina (TCA) son tratamientos de primera línea para la malaria sin complicaciones provocadas por el *Plasmodium falciparum* en muchas zonas endémicas, pero hay poca evidencia de su eficacia en niños anémicos con malaria.

Métodos: La eficacia terapéutica de los tratamientos de 3 días con artesunato-amodiaquina y arteméter-lumefantrina se evaluaron en 437 niños anémico con malaria y 909 niños no anémicos con malaria tras el tratamiento durante un período de siete años (2008-2014). Los patrones de los cambios temporales en los hematocritos fueron clasificados sobre la base de los valores de hematocritos <30% y ⩾30%. La cinética de la disposición de la carencia de hematocritos del 30% después del tratamiento fueron evaluaron usando un modelo no compartimental.

Resultados: La eficacia parasitológica corregida por PCR luego de 28 días del inicio del tratamiento fue significativamente mayor en el artesunato-amodiaquine en comparación con arteméter-lumefantrina en los niños tratados [97% (95%*CI* 92,8 a 100) *versus* 96,4% (95%*CI* 91,3-99,4), *PAG*=0,02], pero fue similar en los niños no anémicos y anémicos. La caída de los parásitos asexuales del tipo hematocritos/100 en las muestras de sangre tomadas fue significativamente mayor en comparación con parasitemias superiores (*P*<0,0001), y en no anémicos en comparación con niños anémicos (*P*=0,007). En los niños anémicos a los cuales fue prescrito, el tiempo medio de recuperación de la anemia (ANRT) fue de 15,4 días (95%*CI* 13,3-17,4) y no ha cambiado con los años. La disminución en el déficit de hematocritos de 30% eran monoexponencial estimando media promedio de tiempo de 1,4 días (95%*CI* 1.2 a 1.6). La anemia de medio tiempo ($t_{½anaemia}$) Correlacionó positivamente con ANRT en los mismos pacientes (r=0,69, *PAG*<0,0001). Los análisis Bland y Altman de múltiplos de 10 $t_{½anaemia}$ y ANRT mostraron un estrecho límite de acuerdo con un sesgo insignificante (*P*=0,07) lo que sugiere que ambos pueden ser utilizados indistintamente en los mismos pacientes.

Conclusiones: El artesunato-amodiaquina y arteméter-lumefantrina permanecen sin complicaciones de tratamientos eficaces *P. falciparum* infecciones en niños nigerianos no anémicos y anémicos en los últimos 7 años de su adopción como tratamientos de primera línea. Estos ACT también pueden conservar los hematocritos a niveles altos de parasitemias y en niños anémicos.

Registro de ensayos: Registro Panafricano de Ensayos Clínicos PACTR201508001188143 3 de julio de 2015; PACTR201510001189370 3 de julio de 2015; PACTR201508001191898 7 de julio de 2015 y PACTR201508001193368, 8 de julio de 2015.

Translated from English version into Spanish by Francisco Gonzalez, through

TRANSLATORS
WITHOUT BORDERS
